# Thymoquinone-induced conformational changes of PAK1 interrupt prosurvival MEK-ERK signaling in colorectal cancer

**DOI:** 10.1186/1476-4598-13-201

**Published:** 2014-08-29

**Authors:** Chirine El-Baba, Vijayalakshmi Mahadevan, Fabian B Fahlbusch, Suma Mohan S, Tilman T Rau, Hala Gali-Muhtasib, Regine Schneider-Stock

**Affiliations:** Experimental Tumorpathology, Institute of Pathology, Friedrich-Alexander University Erlangen-Nürnberg (FAU), Erlangen, Germany; School of Chemical and Biotechnology of the SASTRA University, Thanjavur, India; Department of Pediatrics and Adolescent Medicine, University of Erlangen-Nürnberg, Erlangen, Germany; Department of Biology, American University of Beirut, Beirut, Lebanon

**Keywords:** Thymoquinone, PAK1, ERK1/2, Colorectal cancer, Scaffold function, Kinome analysis, Apoptosis

## Abstract

**Background:**

Thymoquinone (TQ) was shown to reduce tumor growth in several cancer models both *in vitro* and *in vivo*. So far only a few targets of TQ, including protein kinases have been identified. Considering that kinases are promising candidates for targeted anticancer therapy, we studied the complex kinase network regulated by TQ.

**Methods:**

Novel kinase targets influenced by TQ were revealed by *in silico* analysis of peptide array data obtained from TQ-treated HCT116wt cells. Western blotting and kinase activity assays were used to determine changes in kinase expression patterns in colorectal cancer cells (HCT116wt, DLD-1, HT29). To study the viability/apoptotic effects of combining the PAK1 inhibitor IPA-3 and TQ, crystal violet assay and AnnexinV/PI staining were employed. Interactions between PAK1 and ERK1/2 were investigated by co-immunoprecipitation and modeled by docking studies. Transfection with different PAK1 mutants unraveled the role of TQ-induced changes in PAK1 phosphorylation and TQ´s effects on PAK1 scaffold function.

**Results:**

Of the 104 proteins identified, 50 were upregulated ≥2 fold by TQ and included molecules in the AKT-MEK-ERK1/2 pathway. Oncogenic PAK1 emerged as an interesting TQ target. Time-dependent changes in two PAK1 phosphorylation sites generated a specific kinase profile with early increase in pPAK^Thr212^ followed by late increase in pPAK^Thr423^. TQ induced an increase of pERK1/2 and triggered the early formation of an ERK1/2-PAK1 complex. Modeling confirmed that TQ binds in the vicinity of Thr212 accompanied by conformational changes in ERK2-PAK1 binding. Transfecting the cells with the non-phosphorylatable mutant T212A revealed an increase of pPAK^Thr423^ and enhanced apoptosis. Likewise, an increase in apoptosis was observed in cells transfected with both the kinase-dead K299R mutant and PAK1 siRNA. Using structural modeling we suggest that TQ interferes also with the kinase domain consequently disturbing its interaction with pPAK^Thr423^, finally inhibiting MEK-ERK1/2 signaling and disrupting its prosurvival function. pERK1/2 loss was also validated *in vivo*.

**Conclusions:**

Our study shows for the first time that the small molecule TQ directly binds to PAK1 changing its conformation and scaffold function. Because TQ affects the central RAF/MEK/ERK1/2 pathway, the combination of TQ with targeted therapies is worth considering for future anticancer treatments.

**Electronic supplementary material:**

The online version of this article (doi:10.1186/1476-4598-13-201) contains supplementary material, which is available to authorized users.

## Background

Colorectal cancer (CRC) affects yearly more than 1 million people worldwide [1], therefore there is constant need to achieve more effective cures. Over the recent years naturally occurring compounds have received increasing attention because of their anticancer effects [2]. Thymoquinone (TQ), the active compound extracted from *Nigella sativa*, is a very promising anticancer drug, whether used separately or in combination with conventional medicines [2–7]. Interestingly, TQ was found to have only limited toxicity to normal intestinal cells *in vitro* [8] and not to affect the survival or body weight of animals when used at doses up to 25 mg/kg in colorectal cancer animal models [9, 10]. Previous work documented TQ´s growth inhibitory and apoptosis triggering effects on colon and other solid tumors such as uterine sarcoma, breast, and pancreatic cancer in a dose- and time-dependent manner [9, 11–14]. Moreover, TQ was shown to reduce tumor growth and to induce apoptosis in various murine cancer models [5–7, 10, 15, 16]. So far, the anticancer mechanism of TQ is not fully understood; however, several modes of action have been described depending on the stimulus and the cellular context [2].

Protein kinases, the protagonists of phosphorylation, commonly work in complex networks and have become novel candidates for targeted therapy. There are a few studies describing the involvement of TQ in the regulation of kinases i.e. AKT1 [2] , JAK2 [17], JNK [8], IKKb[18], ERK2 [8], CHEK1 [9] andPlk1 [19]. Most of these kinases are frequently deregulated in colon cancer. In the last 10 years several kinase inhibitors have been clinically tested or are currently undergoing clinical trials in targeted therapy [20]. Such therapy blocks the growth and spread of cancer by specifically interfering with molecules affecting tumor cell proliferation and progression. In colorectal cancer we have shown that kinases such as c-Jun N-terminal kinase (JNK) and the prosurvival extracellular-signal-regulated kinase (ERK) were activated under TQ treatment before the onset of apoptosis [8]. The TQ resistance observed in HCT116 p53−/− cells was associated with an upregulation of CHEK1, a serine/threonine kinase cell cycle checkpoint mediator, indicating an inefficient defense mechanism against TQ-induced DNA damage when p53 is absent [9].

So far despite a few reports, the interaction of TQ with the kinase network of colon cancer cells is only poorly understood. Kinase signaling has been extensively investigated in the last decade and new techniques of quantitative proteomics have been developed simplifying the study of complex systems. Thus we performed a quantitative phospho-proteomic analysis to identify new targets in TQ- treated HCT116wt colorectal cancer cells. For the first time we show a direct binding of the plant derived small molecule TQ to the PAK1/ERK1/2 kinase complex leading to conformational changes in protein structures and consequently to apoptosis.

## Results and discussion

### Identification of new TQ targets by peptide array analysis

**Figure 1:**
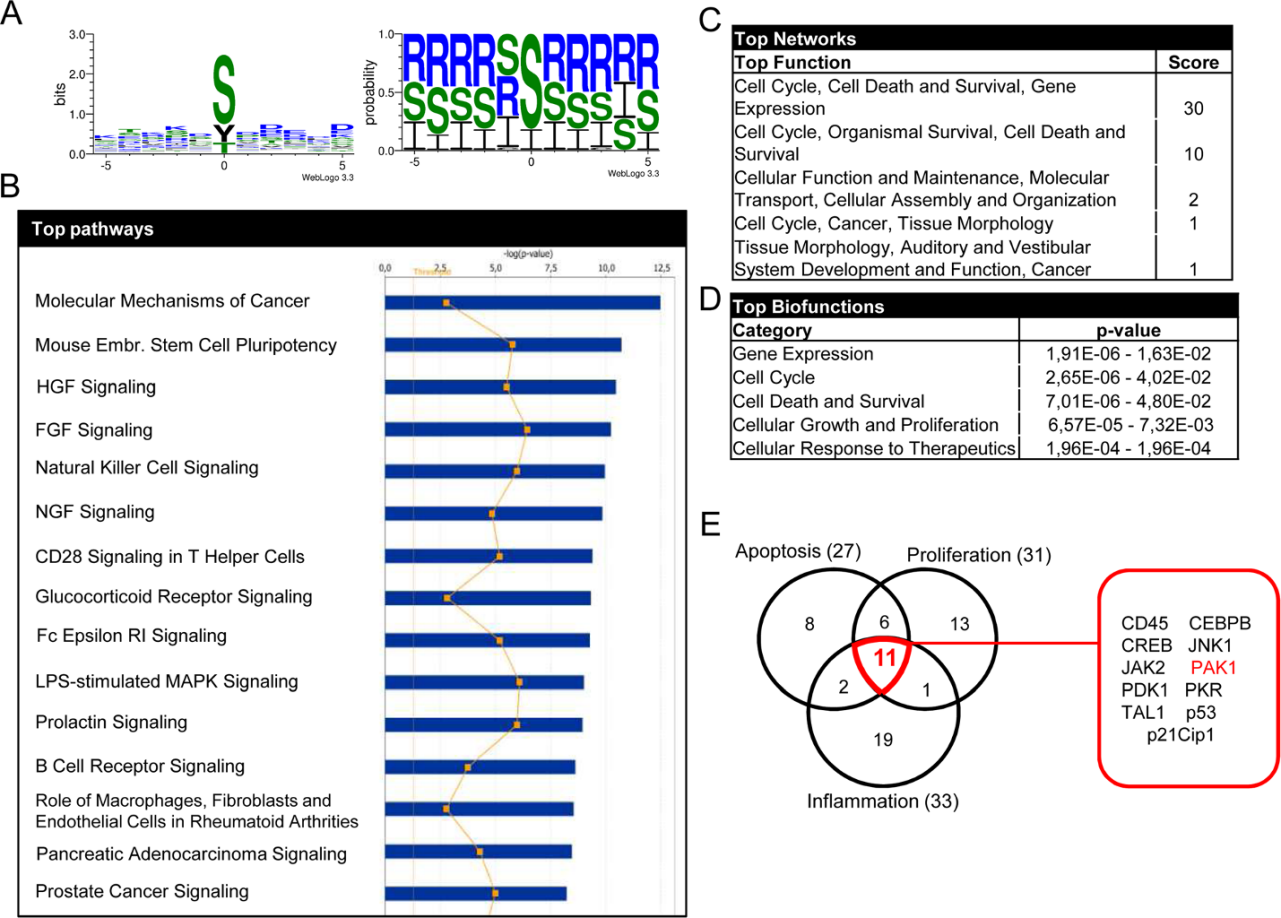
**TQ induces the phosphorylation of a multitude of proteins.** Whole cell lysates of HCT116wt cells treated with 40 μM TQ were probed on PepChip Kinomics v2 peptide array. **A**. Consensus sequence logo obtained, via WebLogo version 3.3, of the identified protein phosphorylation motifs by Pepscan. In the left panel, the phosphorylated amino acid is placed on the position 0 of the x-axis and the frequency of the neighboring amino acids is represented on the y-axis. In the right panel, the amino acids probability is shown according to their polarity (hydrophilic amino acids (R), hydrophobic amino acids (I) and neutral amino acids (S)). **B**. Top canonical pathways identified by IPA analysis system of the candidate proteins. **C**. Function analysis of the different proteins and their scores grouped in networks. **D**. List of top biofunctions involving the identified proteins with p-value <0.05. **E**. Venn diagram of the top 50 candidates (≥2 fold phosphorylation) belonging to one or more of the most relevant cancer related biological functions: apoptosis, proliferation and inflammation. The 11 candidates common to all three groups are listed in the red square.

Kinome analysis allows the characterization and quantification of the phosphorylation profile of a given kinase target under various experimental conditions. Using a peptide array, the treatment of colorectal HCT116wt cells with 40 μM TQ for 24 h led to the identification of 104 proteins with a significant phosphorylation upregulation, among which were 50 proteins and kinases upregulated by ≥2 fold (out of 1152 kinase substrate peptides). Many of these proteins were previously described as TQ targets (such as p21^Cip1^, p53, ERK or NFkB) verifying the feasibility of the array data (Additional file 1: Table S1). The analysis of the phosphorylation motifs of all 104 candidates (Figure 1A) showed that TQ had a propensity for serine phosphorylation as well as an 80% probability to induce phosphorylation of a neutral polar amino acid (S group). *In silico* analysis revealed that the most influenced pathways (Figure 1B) and networks (Figure 1C,1D) were those involved in cancer, cell cycle, cell death, and survival mechanisms. Moreover 24 of the top 50 candidate proteins were grouped into the cancer-related networks “cytoskeleton”, “PI3K/AKT” and “Wnt signaling” (Table 1). The Venn-diagram for the most relevant biological functions identified 11 TQ-modulated proteins that are common in apoptosis, proliferation, and inflammation pathways. Besides the epigenetic marker CEBPB, the analysis included key molecules in the AKT-MEK-ERK pathway, a central signaling network for current targeted therapies (Figure 1E). With a fold-change of 2.12, p21 protein (Cdc42/Rac)-activated kinase 1 (PAK1) was an appealing candidate considering its role in cell growth, invasion, cell migration, cell survival, mitosis and cytoskeletal remodeling of cancer cells [21, 22]. Recently PAK1 was considered a novel subject for targeted cancer therapeutic approaches [23]. Furthermore for the first time, PAK1 has been identified as a TQ target. An extensive pathway mapping of genes differentially phosphorylated by TQ at 24 h and subsequent pathway annotation clustering resulted in 5 significant pathway based clusters. It is interesting that PAK1 was mostly associated with AKT1 and RAF1 (Table 2) in all the pathways in the Annotation cluster3. The top 4 pathways obtained through clustering approach (T-cell receptor signaling, angiogenesis, MAPK and chemokine signaling) also included PAK1 making it the target of our more detailed study of TQ action.

**Table 1:** **Identified TQ targets belonging to cancer-related pathways**

Network	Identified substrate	Phospho site	Prot ID	Folds up (24 h)
**Cytoskeleton**	EphB1	Y594	NP_004432	2.93
	Lamin A/C	S22	CAA27173	2.45
	MAP2v	S1679	NP_114034	2.30
	Syntaxin 1A	S14	NP_004594	2.22
	Lamin B1	S395	NP_005564	2.12
	SNAP23	T24	NP_003816	2.08
**PI3K/AKT**	Lck	S158	AAH13200	3.45
	HSP22	S14	NP_0055180	3.32
	PKR	T451	NP_002750	3.92
	Beta-2-adrenergic receptor-B2AR	S262	NP_000015	2.80
	AKT1	Y326	NP_005154	2.22
	Bone marrow kinase BMX-ETK	Y40	AAC08966	2.16
	Glucocorticoid receptor	S211	NP_000167	2.15
	PAK1	T212	AAC24716	2.12
	Nitric oxide synthase 1	S852	NP_000611	2.09
	CHOP	S79	NP_004074	2.08
	Metabotropic glutamate receptor 1	T695	AAA87843	2.05
	RAF1-c-RAF	S43	NP_002871	2.00
**Wnt**	Beta-catenin	Y142	NP_001895	2.89
	T-cell transcription factor 4	S60	NP_110383	2.59
	N-myc	S263	NP_005369	2.43
	L1 cell adhesion molecule	S1152	NP_000416	2.25
	APC	S2054	NP_000029	2.06
	MAP3K7-TAK1	S192	NP_663304	2.01

**Table 2:** **Pathway mapping of TQ-phosphorylated targets by Annotation cluster3**

Database	Pathway	p-Value	Genes identified by peptide array
KEGG_PATHWAY	T cell receptor signaling pathway	0.001971	PDK1, AKT1, RAF1, PAK1*, MAP3K7
PANTHER_PATHWAY	Angiogenesis	0.008722	AKT1, TCF7L2, APC, RAF1, **PAK1**, CTNNB1, NOS1
KEGG_PATHWAY	MAPK signaling pathway	0.041478	MAX, AKT1, RAF1, **PAK1**, MAP3K7
KEGG_PATHWAY	Chemokine signaling pathway	0.042726	AKT1, CCR2, RAF1, **PAK1**
KEGG_PATHWAY	Renal cell carcinoma	0.053539	AKT1, RAF1, **PAK1**
PANTHER_PATHWAY	Ras Pathway	0.054889	AKT1, RAF1, **PAK1**, MAP3K7
BIOCARTA	Influence of Ras and Rho proteins on G1 to S Transition	0.055681	AKT1, RAF1, **PAK1**
KEGG_PATHWAY	ErbB signaling pathway	0.079412	AKT1, RAF1, **PAK1**
KEGG_PATHWAY	Focal adhesion	0.091138	AKT1, RAF1, **PAK1**, CTNNB1
KEGG_PATHWAY	Fc gamma R-mediated phagocytosis	0.093575	AKT1, RAF1, **PAK1**
BIOCARTA	MAPKinase Signaling Pathway	0.094598	MAX, RAF1, **PAK1**, MAP3K7
PANTHER_PATHWAY	Inflammation mediated by chemokine and cytokine signaling pathway	0.229979	AKT1, CCR2, RAF1, **PAK1**, MAP3K7
PANTHER_PATHWAY	T cell activation	0.27141	AKT1, RAF1, **PAK1**

*PAK1 is given in bold letters.

### TQ effect on PAK1 phosphorylation

**Figure 2:**
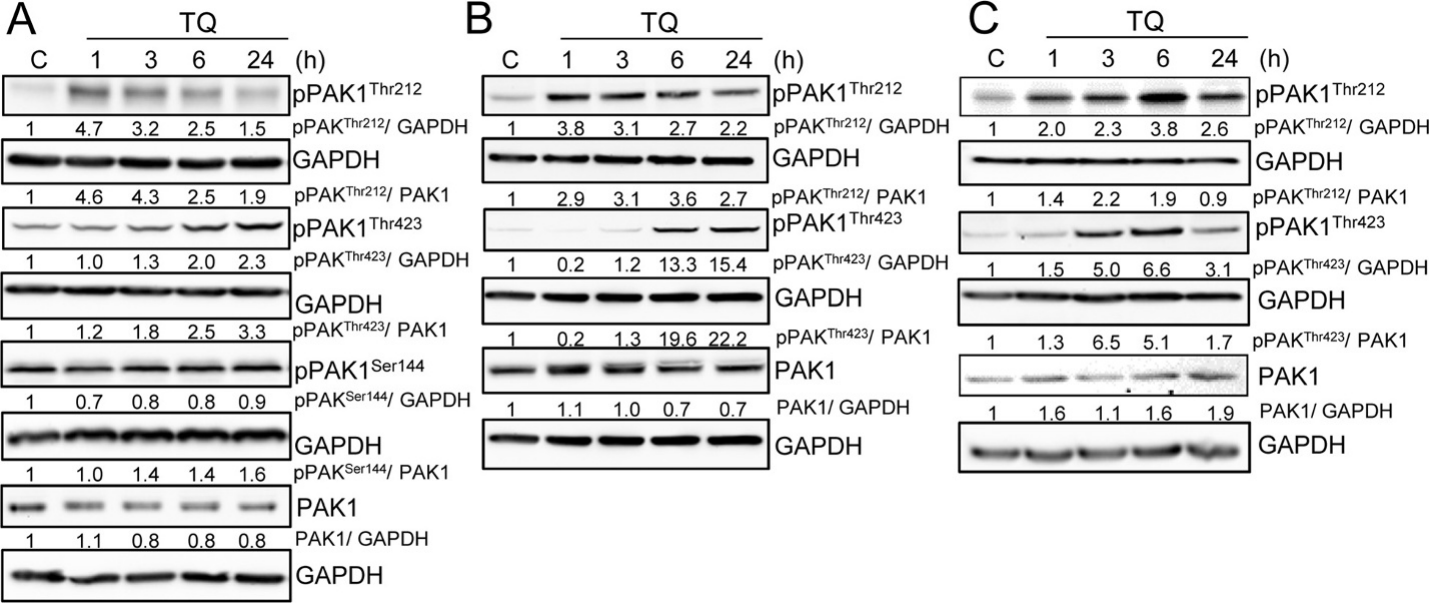
**TQ induces the phosphorylation of pPAK** ^**Thr212**^ **and pPAK** ^**Thr423**^ **in several colorectal cell lines.** HCT116wt **A**, DLD-1 **B**, and HT29 **C**. cells were treated with 40 μM TQ and collected after 1, 3, 6 and 24 hours. Untreated cells were used as control. Whole cell lysates were immunoblotted against total PAK1, pPAK^Thr212^, pPAK^Thr423^ and pPAK^Ser144^ (only for HCT116wt). Blots were then probed with GAPDH for loading control. Data shown are representative of two independent experiments.

In HCT116wt cells, the TQ-induced activation of pPAK1^Thr212^ observed in the array analysis was verified by western blotting (Figure 2A). Although PAK1 has several different phosphorylation sites, only PAK1^Thr212^ was spotted on the PEPSCAN peptide array. In addition, we investigated two other well-studied phosphorylation sites: pPAK1^Thr423^ and pPAK1^Ser144^, by western blotting. Time-dependent changes in the expression patterns of pPAK1^Thr212^ and pPAK1^Thr423^ generated a specific kinase profile at early and late time points after TQ exposure. While pPAK1^Thr212^ showed a fast induction at 1 and 3 h, pPAK1^Thr423^, found within the activation loop of PAK1, increased at 24 h (Figure 2A). In contrast, pPAK1^Ser144^ and total PAK1 levels did not change over time (Figure 2A). Investigating another colorectal cancer cell line, DLD-1 showed the same profile of pPAK1^Thr212^ and pPAK1^Thr423^ regulation over time (Figure 2B). As expected, the TQ resistant HT29 cells [8] showed a different kinase profile where both pPAK1^Thr212^ and pPAK1^Thr423^ levels increased at the same time from 3 to 6 h and decreased at 24 h (Figure 2C).

### TQ is a possible PAK1 inhibitor

To further understand the role of PAK1 in TQ-induced apoptosis, we used 1,1′-disulfanediyldinaphthalen-2-ol (IPA-3), the allosteric inhibitor of PAK1. IPA-3 prevents pPAK1^Thr423^ phosphorylation by targeting the auto-regulatory site and disrupting the functional interaction between PAK1 and Cdc42 in addition to inhibiting the activity of other kinases [23, 24]. Here we show that TQ did not change the activity of AKT2, GSKα, GSKβ and p38, suggesting that this molecule might be more specific than IPA-3 in interacting with PAK1 (Additional file 2: Figure S1). In addition, we performed a crystal violet viability assay and obtained an IC_50_ of 141 μM ±30.7 in normal intestinal epithelial HCEC cells in comparison to an IC_50_ of 50 μM ± 6 in HCT116 tumor cells (Additional file 3: Figure S2), thus confirming the limited toxicity of TQ to normal cells.

**Figure 3:**
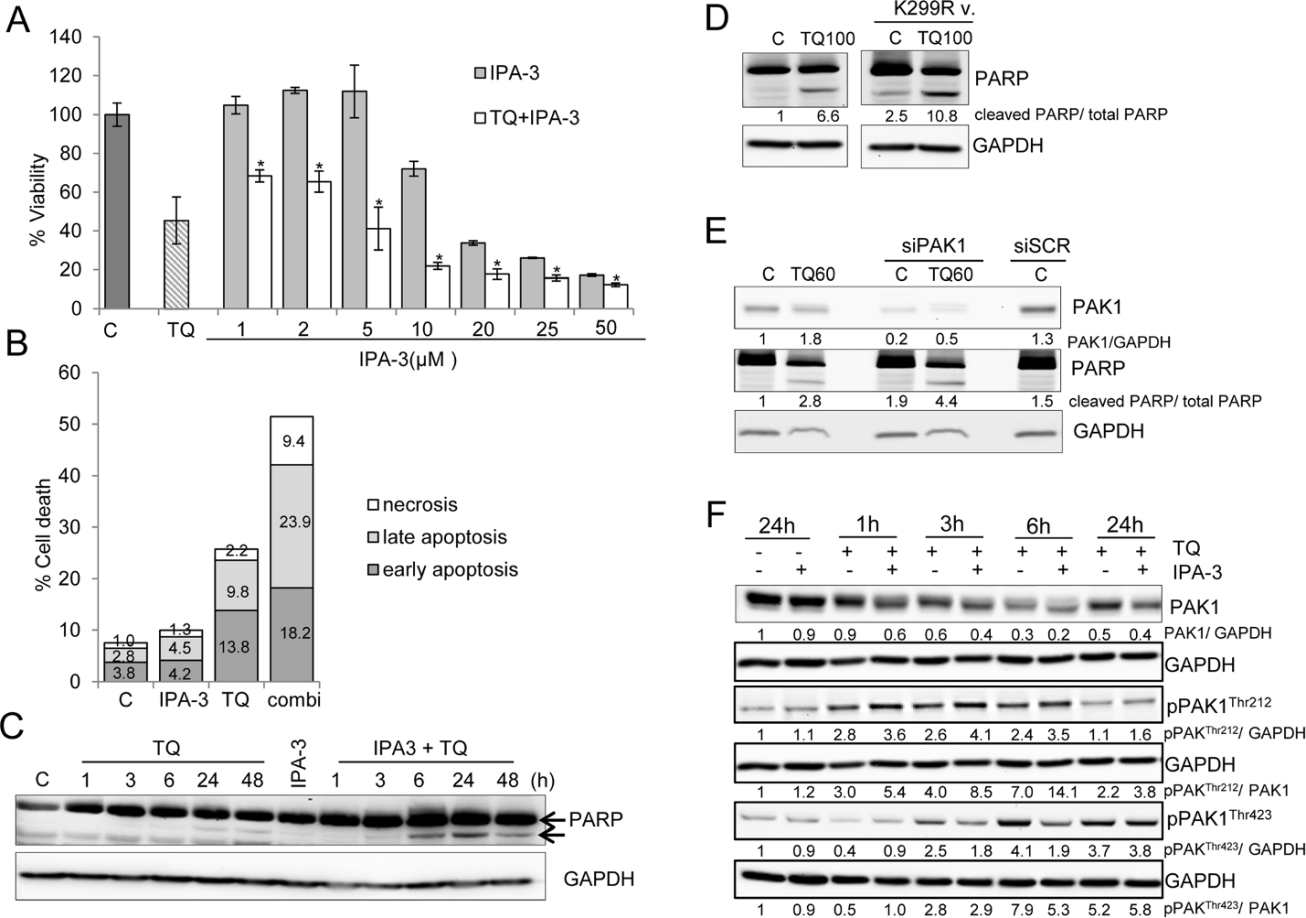
**Combining TQ and the PAK inhibitor IPA-3 increases apoptosis. A**. HCT116wt cells, pretreated with different concentrations of IPA-3 (1-50 μM) alone or with TQ 40 μM for 24 hours, were stained with crystal violet dye and assessed for viability. Data are presented as percentage of control. *indicates a p-value < 0.001 when comparing IPA-3 treatment to IPA-3 + TQ treatment. Each value is the mean ± SD of three independent experiments each done in quadruplicates. **B**. Cells pretreated with 10 μM IPA-3 for one hour followed by 24 hours incubation with 40 μM TQ were collected and analyzed by flow cytometry for a double AnnexinV/PI staining. The percentage of the different cell populations was calculated by FlowJo software. **C**. Cells were pretreated for 1 hour with 10 μM IPA-3 and then incubated with 40 μM TQ. Cell pellets were collected after 1, 3, 6, 24 and 48 hours. Lysates were blotted against PARP and GAPDH for loading control. **D**. PARP cleavage was detected in endogenous (left blots) and kinase-dead dominant negative PAK1 K299R mutant transfected HCT116 wt cells (right blots) after 24 hours of 100 μM TQ. Higher TQ concentration was used in all transfection experiments due to the high cell density. **E**. Cells were transfected with PAK1 siRNA or scrambled (SCR) siRNA and checked for PAK1 knockdown by western blotting. PARP cleavage was detected in endogenous and PAK1 siRNA transfected cells after 24 hours of 60 μM TQ. SCR siRNA was used as transfection control. GAPDH was used as loading control. **F**. Cell lysates were immunoblotted against pPAK^Thr212^, pPAK^Thr423^ and total PAK1. GAPDH was used as a loading control. Data shown are representative of two independent experiments.

Since IPA-3 does not inhibit already activated PAK1, HCT116wt cells were pre-incubated with IPA-3 for 1 h and then stimulated with 40 μM TQ (Figure 3). Crystal violet staining showed that 30% of the cells were dead after 10 μM IPA-3 (Figure 3A). Interestingly, the combination of TQ and IPA-3 (10 μM) caused significantly more cell death and decreased cell viability by 70% (Figure 3A). AnnexinV/PI staining revealed that most dead cells were in late apoptosis (Figure 3B). TQ induced the “apoptotic” cleavage of PARP (89 kDa fragment) at 24 h, whereas in response to IPA-3 the cleavage was stronger and occurred earlier at 6 h (Figure 3C). Furthermore, transfection with kinase-dead dominant negative K299R mutant led to a larger increase in PARP cleavage showing more apoptosis when the kinase activity is lost (Figure 3D). Similarly, PAK1 siRNA transfection of cells followed by TQ treatment induced an increase in the pro-apoptotic response (Figure 3E).

In a next step, we determined changes in the expression of the different phosphorylation sites of PAK1 in IPA-3 and/or TQ treated cells (Figure 3F). As expected, pPAK1^Thr423^ protein level decreased when IPA-3 was combined with TQ at 3 and 6 h but this decrease was not sustained at 24 h. To further understand this finding, we modeled the interaction between IPA-3, PAK1 and TQ. Docking IPA-3 to PAK1 (Additional file 4: Figure S3A) revealed that IPA-3 binds to the CRIB motif (75–90) in the autoregulatory region of PAK1 dimer in a mode that enhances the interaction between both monomers (binding site first monomer: His83 and Gly98, second monomer: Met99). When TQ is docked into the IPA-3-bound conformation, IPA-3 still binds with both monomers. TQ also interacts with residues of both PAK1 monomers in the auto-inhibited dimer conformation (Additional file 4: Figure S3B and Additional file 5: Table S2). In this binding mode, TQ interaction with the kinase inhibitory segment of PAK1 (Asn38 residue) possibly causes the masking of the PAK1 activation loop and prevents the action of IPA-3 on Thr423 (Additional file 4: Figure S3).

The phosphorylation of pPAK1^Thr212^ in TQ and IPA-3 co-treated cells showed a remarkable increase in comparison to TQ alone (Figure 3F). This was surprising considering that IPA-3 only interrupts the interaction between Cdc42 and the autoregulatory site at Thr423 residue of PAK1 [24, 25]. Knowing that pPAK1^Thr212^ is a major target of ERK2 [26], we further studied the role of ERK1/2/PAK1 interaction in response to TQ.

### TQ triggers conformational changes in PAK1 and induces ERK1/2-PAK1 binding

**Figure 4:**
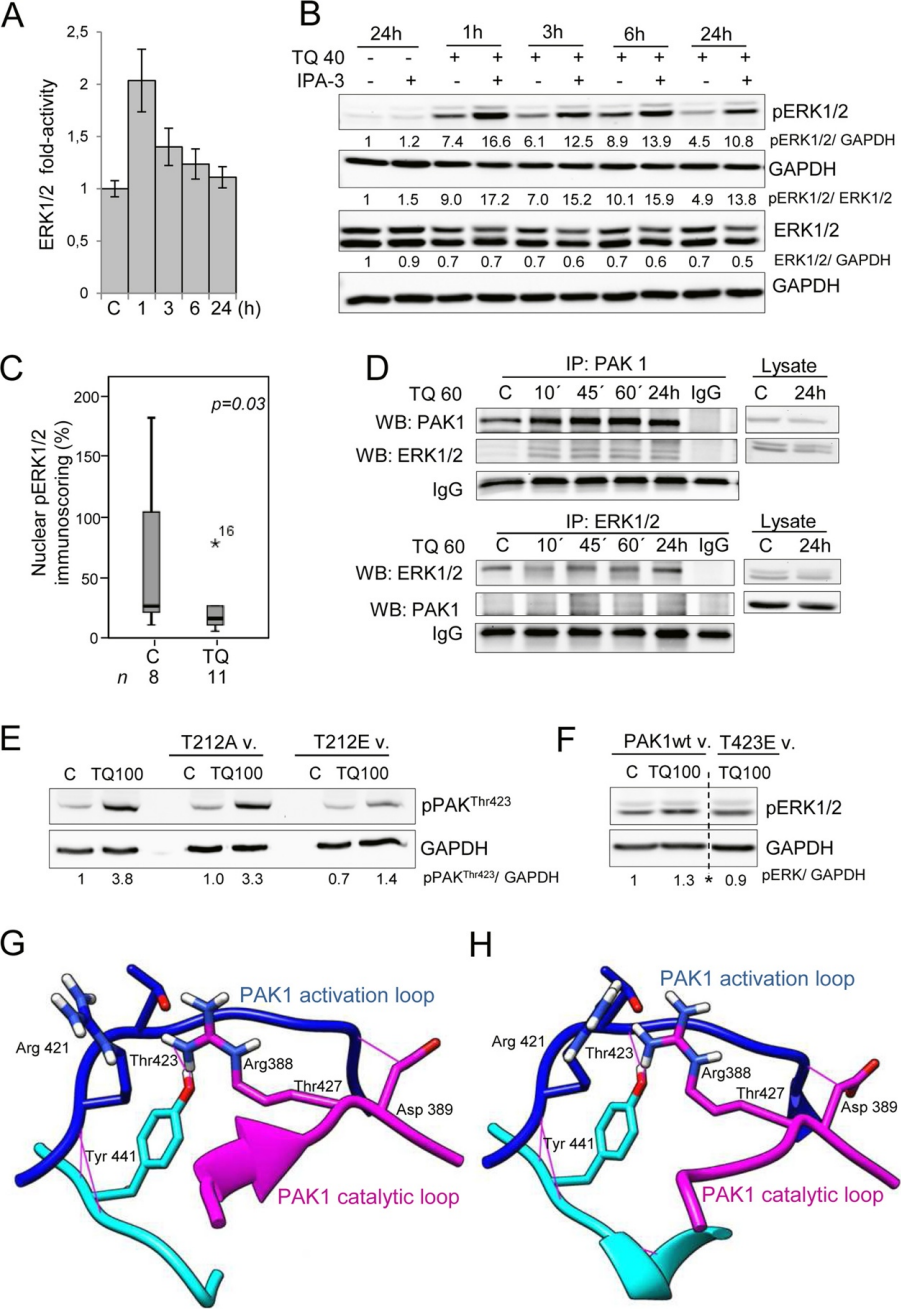
**TQ induces early upregulation of ERK1/2 and the formation of a PAK1-ERK1/2 complex. A**. TQ treated cells were assessed for ERK1/2 activity. Data is presented as fold activity normalized to control activity. Each value is the mean ± SD of two independent experiments each done in duplicates. **B**. Cells pretreated for 1 hour with 10 μM IPA-3 were incubated with 40 μM TQ and cell pellets were collected after 1, 3, 6 and 24 hours. Lysates were immunoblotted for pERK1/2 and total ERK1/2. GAPDH was used as loading control. **C**. Box plot analysis of the percentage of tumor cells expressing nuclear pERK1/2 in mouse xenografts after TQ treatment. n represents the number of investigated mouse tissues in each group. *5 corresponds to a sample outlayer. **D**. Cell lysates stimulated with 60 μM TQ for 10, 45 minutes, 1 and 24 hours were immunoprecipitated for PAK1 (upper panel) and ERK1/2 (lower panel) and blotted against total PAK and ERK1/2. IgG bands were used as loading control. Data shown are representative of two independent experiments. **E**. Cells transfected with the PAK1wt vector and the T423E mutant vector were exposed to 24 hours of 100 μM TQ. 30 μg of proteins was immunoblotted against pERK1/2. *indicates the omission of intermediate samples. **F**. Cells transfected or not with T212A and T212E mutants of PAK1 were treated with 100 μM TQ. Lysates were immunoblotted against pPAK^Thr423^. Data shown are representative of two independent experiments. **G**. Model of PAK1 activation (in dark blue) and catalytic loop (purple) presenting the different amino acids of interest. The kinase domain in PAK1 (residues 249–545) is where T423 resides. **H**. Model of PAK1 activation (in dark blue) and catalytic loop (purple) in the presence of TQ showing rearrangements in the main activation loop under TQ. There are additional hydrogen bonds in the catalytic loop region involving residues Arg388 and Arg421 which are known to interact with Thr423 for the catalytic activity.

In a next step we investigated the effect of TQ on ERK1/2 in HCT116wt cells. Kinase activity assay revealed that ERK1/2 activity significantly increased after 1 h of TQ treatment (Figure 4A). This was confirmed by western blotting, where pERK1/2 level was elevated at the same time point followed by a later decrease to normal levels (Figure 4B). In addition to the previously published increase in TUNEL and cleaved caspase-3 [10], immunohistochemical staining of the prosurvival pERK1/2 was significantly (p < 0.05) lost in the nuclei of the TQ treated tumor xenografts (Figure 4C and Additional file 6: Figure S4).

**Figure 5:**
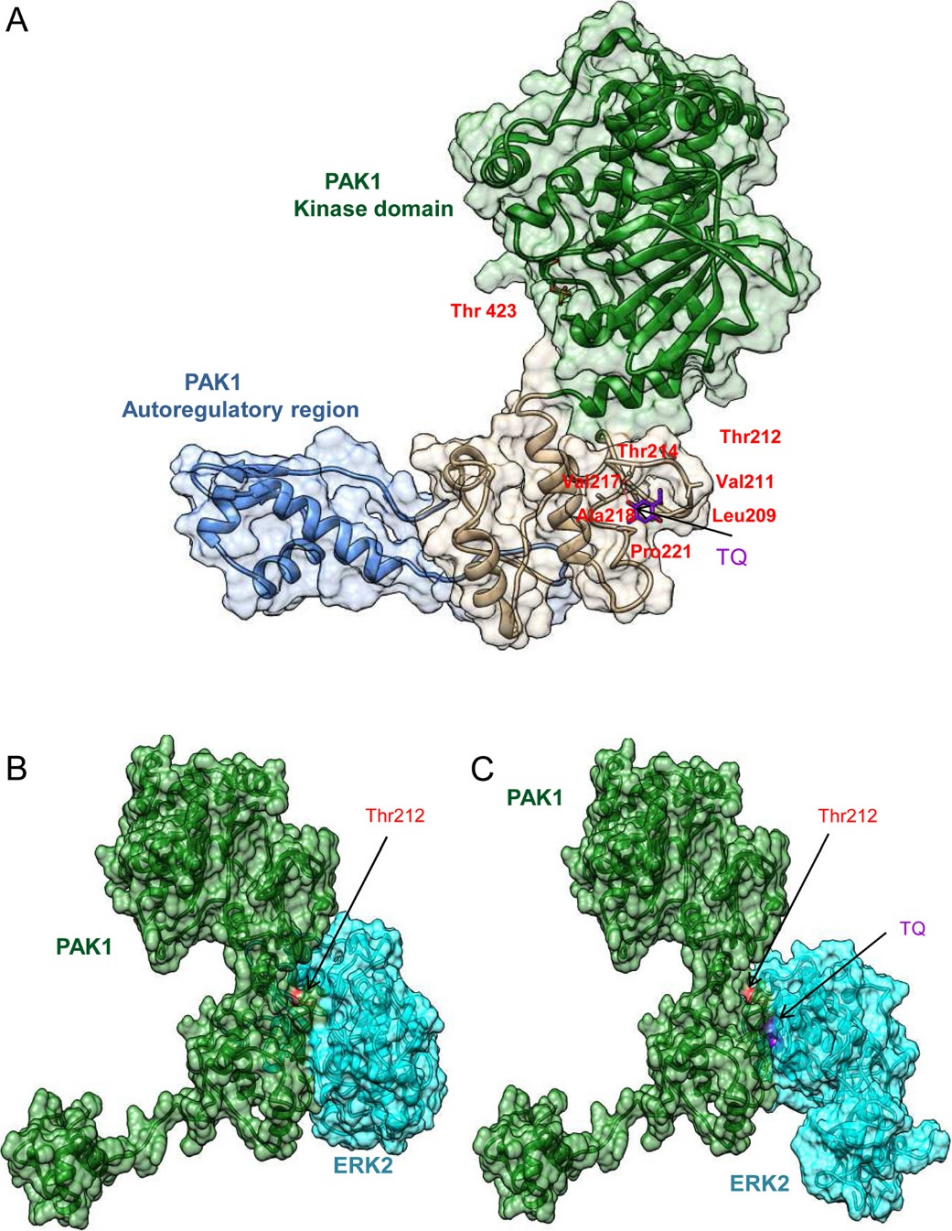
**TQ binds to PAK1 in the vicinity of Thr212. A**. Surface representation of possible active conformation of PAK1 bound to TQ in the vicinity of pPAK1Thr212. TQ is represented in stick (purple). PAK1 residues within the 4 Å interface of the binding site are labelled in red. The kinase domain and the regulatory domain of PAK1 are marked in green and blue respectively. **B**. Surface representation of the PAK1-ERK2 complex. PAK1 and ERK2 are shown in green and cyan respectively. Position of Thr212 is highlighted in red. **C**. Surface representation of PAK1-ERK2 complex in the presence of TQ. Positions of Thr212 and TQ are highlighted in red and purple respectively.

Furthermore, time course analysis of pPAK^Thr212^ and pERK1/2 levels at early time points revealed that pPAK^Thr212^ increase can be observed as early as 30 min whereas pERK1/2 increase started after 45 min of TQ treatment (Additional file 7: Figure S5). This suggests an involvement of other kinases in the phosphorylation of PAK1 at the Thr212 site in response to TQ. Interestingly, co-immunoprecipitation studies showed that TQ induced the formation of a PAK1 and ERK1/2 complex (Figure 4D). To better understand the nature of ERK1/2/PAK1 interaction in response to TQ, we modeled the PAK1-TQ interaction by docking PAK1 and TQ and identified a possible ligand binding site at the vicinity of Thr212 (Figure 5A) with a docking score of −2.316 (Additional file 5: Table S2) [27–30]. The energy of ERK2 bound to PAK1 conformation changed in the presence of TQ from -21883 kcal/mol to −22076 kcal/mol suggesting that TQ strengthens ERK2 binding to PAK1 [31]. It may also be noted from Figure 5B and Figure 5C that ERK2 binds in a different mode to PAK1 in the presence of TQ. ERK2 binding to a different conformation of PAK1 may prevent ERK2 from phosphorylating the Thr212 of PAK1. This could explain why pPAK1^Thr212^ levels decrease over time in response to TQ (Figure 2A). Furthermore, TQ-induced pERK1/2 levels were not reduced by IPA-3 treatment, instead ERK1/2 activity was further enhanced from 1 h to 6 h when TQ was combined with IPA-3 (Figure 4B), suggesting the involvement of other upstream kinases in phosphorylating ERK1/2. If the TQ-triggered closer binding between ERK1/2 and PAK1 is disrupted under IPA-3 this might explain the slight increase in pPAK1^Thr212^ levels when TQ is combined with IPA-3 (Figure 3F).

**Figure 6:**
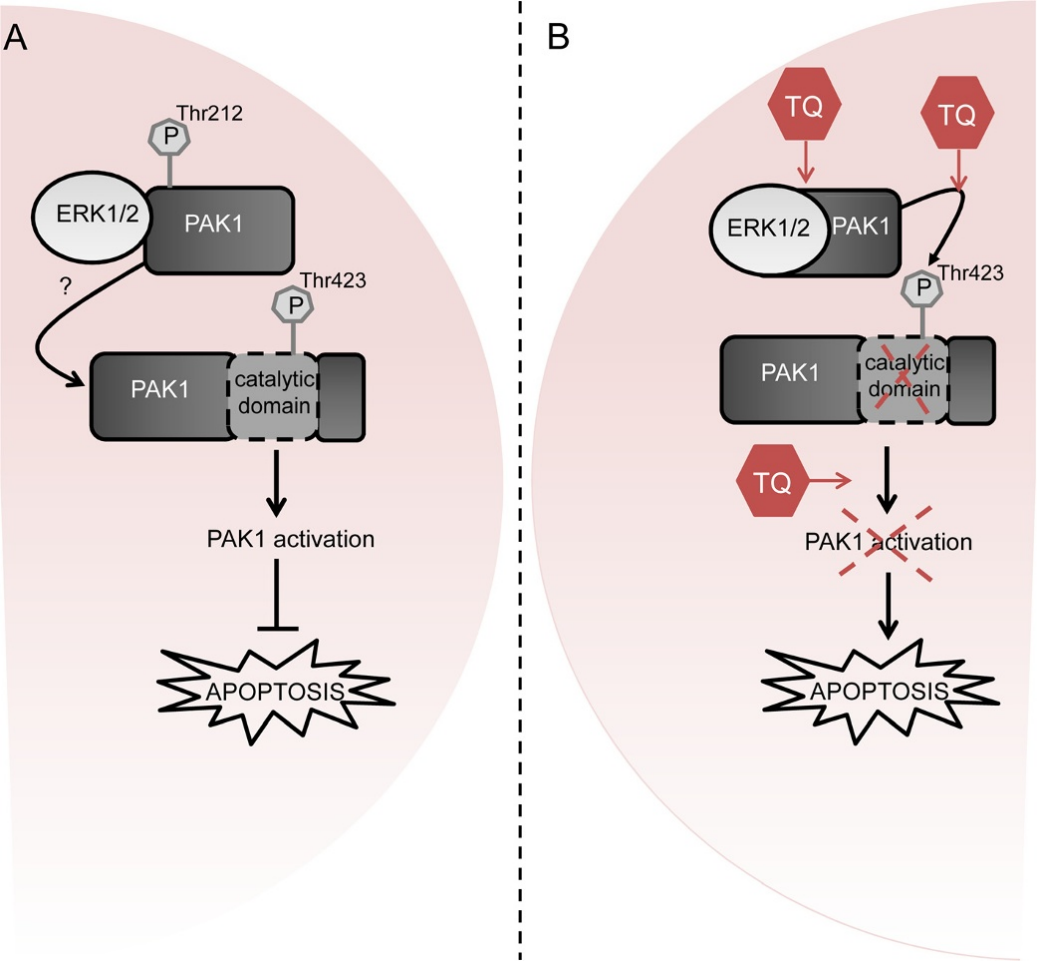
**TQ induces changes in the PAK1-ERK1/2 complex conformation inhibiting the prosurvival role of PAK1. A**. *Without TQ*, in cancer cells, ERK2 phosphorylates pPAK1^Thr212^ (I). While an interaction between pPAK1^Thr212^ and pPAK1^Thr423^ is so far not reported (II), pPAK1^Thr423^ is known to induce the catalytic activity of PAK1 (III) thus leading to the activation of the prosurvival ERK1/2 pathway (IV). **B**. *Upon TQ* we observed massive conformational changes of PAK1 disrupting its scaffold function in prosurvival PAK1/MEK/ERK1/2 signaling and leading to the following modified signaling: ERK1/2-PAK1 binding is reinforced preventing pPAK1^Thr212^ phosphorylation by ERK1/2 (I). This leads to an increased phosphorylation at the Thr423 site (II) which impairs the interference with the catalytic domain of PAK1 and prevents PAK1 activation (III) finally resulting in apoptosis induction (IV).

From these findings we propose a functional relation between both phosphorylation sites of PAK1. Indeed, T212A mutant induced a significant increase in pPAK1^Thr423^ levels whereas the hyperphosphorylated T212E mutant showed a lesser extent of increase (Figure 4E). As presented in Additional file 5: Table S2, TQ binds in the vicinity of Thr212 and no binding occurs next to the 423 site. Therefore we speculate that the chain reaction orchestrated by TQ starts at Thr212 site, which correlates with the western blot pattern (Figure 2A). Furthermore, T423E resulted in a decrease in ERK1/2 phosphorylation (Figure 4F) suggesting an impaired interference of Thr423 residue with the kinase domain of PAK1 under TQ treatment. We analysed the structural changes induced by TQ on PAK1 catalytic site and activation loop. Figure 4G and Figure 4H depict the structural conformation of the catalytic domain of PAK1 in the absence (Figure 4G) or presence of TQ (Figure 4H). The kinase domain shows a root-mean-square deviation of 0.38 Å accompanied by rearrangements in the main activation loop. Hydrogen bond analysis (Additional file 8: Table S3) showed that a higher number of hydrogen bonds are formed in the catalytic loop region in the presence of TQ involving residues Arg388 and Arg421 which are known to interact with Thr423 for the catalytic activity. Finally this results in a disturbed interaction between the Thr423 site and the catalytic kinase domain which inhibits the PAK1 kinase activity and its prosurvival signaling (Figure 6). There are three other lines of evidence for this hypothesis: first, early TQ-induced ERK1/2 activation is inhibited when PAK1^Thr423^ is maximally phosphorylated at 24 h. Second, when IPA-3 is combined with TQ there is a decrease in Thr423 phosphorylation at early time points accompanied by a significant upregulation of pERK1/2 levels that confirms an early activation of MEK-ERK signaling. The lack of inhibition of pPAK1^Thr423^ at 24 h is closely associated with a decrease in prosurvival ERK1/2 activation and enhanced apoptosis induction at 24 h. This can be explained by another structural modeling showing that TQ has the ability to effectively bind to the autoregulatory domain of PAK1 preventing IPA-3 ability to interrupt the interaction between Cdc42 and pPAK1^Thr423^. Third, the pPAK1^Thr212^ upregulation is not as dramatic as the pERK1/2 activation after combined TQ and IPA-3 treatment reinforcing the inhibitory loop for PAK1 activation. Although pERK1/2 should disappear completely by the higher level of pPAK1^Thr423^ at 24 h, it is worth mentioning that other secondary kinase loops could be at play. Consequently the phosphorylation status of Thr423 and PAK1 kinase activity is affected by TQ proving the experimental observations reported here.

## Conclusions

PAK1 has been described as a scaffold for ERK1/2/MEK to recruit MEK to RAF at the membrane [32] thus facilitating signaling through the ERK1/2 pathway [26, 33]. We have shown previously a prosurvival function of ERK1/2 under TQ, since inhibition of ERK1/2 by the MEK inhibitor PD98059 resulted in enhanced apoptosis [8]. In the present study and for the first time we document that a small natural molecule, TQ, directly binds to PAK1/ERK kinase complex, induces considerable conformational changes of PAK1 and interrupts its scaffold function. Upon TQ the interference of Thr423 phosphorylation with the kinase domain is disrupted inhibiting the PAK1 kinase activity and its prosurvival signaling [23]. TQ-induced loss of ERK1/2 dependent phosphorylation at Thr212 of PAK1 provides another negative feedback of prosurvival PAK1/MEK/ERK1/2 signaling. The loss of nuclear pERK1/2 in TQ-treated mouse xenografts confirms the *in vivo* relevance of our finding. Overall, our approach allowed an identification of several novel TQ targets which could be used in the future to evaluate the therapeutic benefit of combination therapies.

## Methods

### Cell lines and reagents

Human colon carcinoma HCT116wt, DLD-1 and HT29 cells were grown in RPMI 1640 medium (PAA Laboratories GmbH, Pasching Austria) supplemented with 10% fetal bovine serum (FBS) and 1% penicillin-streptomycin (PANTM Biotech GmbH, Germany) and kept at 37°C in a humidified incubator (95% air, 5%CO2). Cells were treated at 50% confluency with 40 or 60 μM TQ (Sigma-Aldrich) dissolved in DMSO (final concentration less than 0.1%) and collected at different time points. PAK1 inhibition was performed by preincubating the cells with 10 μM IPA-3 (Sigma-Aldrich) for 1 hour.

### Peptide Array PepChip Kinomics v2

HCT116wt cells (1.5x10^5^/ml) were treated with 40 μM TQ and collected after 24 hours. Proteins were extracted and shipped to manufacturer (Pepscan, Netherland) according to manufacturer protocol. Briefly, cell pellets were lysed (20 mM Tris–HCl (pH 7.5), 150 mM NaCl, 1 mM EDTA, 1 mM EGTA, 1% Triton X-100, 2.5 mM sodium pyrophosphate, 1 mM beta-glycercophosphate, 1 mM sodium orthovanadate (Na3VO3), 1 mM NaF, Roche Complete protease inhibitor cocktail). After sonication, the lysates were spin down and snap frozen before being shipped to the manufacturer. The concentration of the samples was determined using Bradford Protein Assay (Bio-Rad, Hercules, CA). Samples were then hybridized on PepChip Kinomics v2 chip and the kinome profiling was performed by the Pepscan Company. The peptide array was done with samples obtained from two independent experiments and every sample was spotted two times on the array.

### Array Analysis software Ingenuity pathway analysis (IPA)

Peptide array data was analyzed by Ingenuity pathway analysis software (IPA, version 9.0, Ingenuity © Systems, http://www.ingenuity.com, Mountain View, CA, USA). The data set contained upregulated proteins (fold-change ≥ 2 after 24 hours of 40 μM TQ treatment) identified by the pepscan peptide array and their protein ID number. Statistical significance of the different pathways obtained by IPA was calculated using a right-tailed Fisher’s Exact test [34]. Pathways with a p–value ≤ 0.05 were selected. WebLogo v3 application (http://weblogo.threeplusone.com/) was used to analyze the phosphorylation consensus motifs of all the kinases and proteins identified by the array.

### Pathway Annotation Clustering

The probable pathways by which the 50 candidate proteins, with fold-change ≥ 2 after 24 hours of 40 μM TQ treatment, could interfere were predicted using DAVID bioinformatics tool (http://david.abcc.ncifcrf.gov) [35, 36]. For pathway mapping, Uniprot IDs of differentially phosphorylated proteins were submitted to the DAVID web resource with colorectal cancer specific proteins from Human Protein Atlas (http://www.proteinatlas.org/) as background. Pathway predictions were made by including the subset pathway databases: KEGG, Panther, Biocarta, Reactome and BBID followed by pathway annotation clustering.

### Western blotting

HCT116wt, DLD-1 and HT29 cells were grown to 50% confluency and treated with 40 μMTQ and harvested over different time points. The cells were then lysed with RIPA lysis buffer (150 mM NaCl, 0.5% DOC, 0.1% SDS, 1% NP40, 50 mM Tris pH8) containing a cocktail of protease inhibitors. The protein concentration of all samples was determined by by DC BioRad protein assay kit (BioRad Laboratories, Hercules, CA) using bovine serum albumin as standard. Total proteins (50 μg /sample) were separated on an 8–12% SDS polyacrylamide gel and transferred to nitrocellulose membrane by blotting. After blocking with 5% non-fat dry milk in TBST buffer, membranes were incubated with primary antibody at 4°C overnight, washed three times, with TBST buffer, and incubated again with the corresponding HRP-conjugated secondary antibody at room temperature for 1 h. The membranes were then washed with TBST buffer and protein bands were detected by enhanced chemiluminescence. PAK1, ERK1/2, pERK1/2 and PARP were purchased from Cell signaling; pPAK^Thr212^ and pPAK^Ser144^ from Abcam and pPAK^Thr423^ from Abgent. Densitometric analysis was performed for all western blots using ImageJ 1.45 s software.

### Immunoprecipitation

Cells treated with 60 μM TQ for 10, 45, 60 minutes and 24 hours were collected and lysed using RIPA buffer supplemented with proteases and phosphatases inhibitors. Immunoprecipitiation was performed using the Dynabeads Protein G magnetic separation KIT as per manufacturer protocol (Invitrogen). After incubating the beads with 600 μg of proteins, they were incubated with anti-PAK1 and anti-ERK1/2 antibodies. The precipitated lysates were then loaded into SDS-PAGE gels and immunoblotted against total PAK1 and ERK1/2.

### Plasmids and siRNA transfections

Plasmids (PAK1wt, K299R, T212E, T212A, T423E) were a gift from Prof. Jonathan Chernoff (Fox Chase Cancer Center, 333 Cottman Avenue, Philadelphia). Cells seeded in 6-well plates were transfected at 90% confluency with 1 μg of the different plasmids for 6 hours, using Invitrogen Lipofectamine 2000 according to manufacturer instructions. Afterwards cells were treated with 100 μM TQ (due to the high cellular density) and collected after 24 hours. Cell lysates were then used for Western blotting analysis.

PAK1 and scrambled siRNA reagents were purchased from Thermo Fisher Scientific (Dharmacon RNAi Technologies). Lipofectamine RNAiMAX reagent was used to transfect HCT116 cells with 10 μM siRNA, according to manufacturer’s instructions. The next day the transfected cells were split and 50% confluent plates were treated with 60 μM TQ. Cell lysates were then used for Western blotting analysis.

### Structural analysis by docking

To understand the interaction between PAK1 and TQ, we modeled the structure of PAK1 and performed molecular docking with TQ using the Schrodinger suite (Maestro, version 9.3, Schrodinger, Inc, New York, NY, 2012). A model of the possible active state conformation of PAK1 was obtained by integrating the available crystal structures of the autoinhibitory domain 78-147(PDB ID: 1F3M) [27] and the kinase domain 250-542(PDB ID: 3Q52) [28] of PAK1 and a threaded model of the region corresponding to 148–249 using ITASSER [29, 30]. The possible ligand binding sites in PAK1 was analyzed using the SiteMap module (SiteMap, version 2.6, Schrödinger, Inc., New York, NY, 2012) of the Schrodinger suite. In order to analyze the binding mode of TQ near to Thr212 site, grids were generated focusing on Thr212 site and the interacting residues were identified. To study the interaction details of PAK1 and ERK2, the crystal structure of ERK2 available with PDB ID:2ERK [31] was docked to the TQ bound and unbound conformations of PAK1 using ClusPro [37, 38]. The energies of the complexes were determined through energy minimization using AMBER11 [39] . Crystal structure of the autoinhibited dimer of PAK1 (PDB ID: 1F3M) was used to understand the binding mode of IPA-3 on PAK1 [29]. The structural coordinates of the residues (416–422), missing in the PDB, belong to the kinase activation segment of PAK1 and were modeled using Modeller9v12 [40] . IPA-3 was then docked to the autoregulatory region of PAK1 (autoinhibited conformation). To analyze the combined effect of TQ and IPA-3 on PAK1, TQ was docked to the IPA-3 bound conformation of PAK1. All the dockings were performed using the extra precision mode of the Glide program of Schrodinger (Glide, version 5.8, Schrödinger, Inc., New York, NY, 2012). The structures of TQ and IPA-3 were obtained from the PubChem database. Protein and ligand structures for docking were prepared using Protein Preparation Wizard and LigPrep utilities of Schrodinger (LigPrep, version 2.5, Schrödinger, Inc., New York, NY, 2012). All renderings were done using Chimera 1.8 [41].

### Kinase Activity assay

Multi-kinase ELISA array was performed as per the manufacturer protocol (Symansis). Briefly, Cells treated with 40 μM TQ were collected after 1, 3, 6, 24 hours and lysed with 1X denaturing cell lysis buffer (Symansis CLB001). 30 μg of proteins were loaded on pre-coated strips with antibodies corresponding to the investigated kinases. After several washing and hybridization steps, absorbance of each well was measured at 450 nm using VICTORTM X3 multilabel reader.

### Cell Viability assay

Cellular viability was measured by crystal violet staining. HCT116wt (3.75 × 10^4^/ml) cells were seeded in 96 well plate and treated with 40 μM TQ and/or 1-50 μM IPA-3 for 24 hours. The treated cells were washed once with PBS, fixed for 15 min in a crystal violet solution (0.5% crystal violet in 20% methanol) at room temperature, then washed twice with water and air-dried. The stained cells were solubilized with methanol for 15 min with mild agitation. Absorbance was measured at 595 nm using VICTORTM X3 multilabel reader. IC_50_ values were calculated using EXCEL 2010. Statistical analysis was performed using SPSS version 19. One tailed Student T-Test was performed by comparing 2 samples assuming that they have equal variances.

### Annexin V/Propidium Iodide staining

Apoptosis was measured using Annexin V/PI co-staining. HCT116wt cells (1.5 × 10^5^/ml) were pretreated with IPA-3 (10 μM) for 1 hour then treated with TQ (40 μM) for 24 hours. After collection, cells were centrifuged at 200 g for 5 min, 4°C and washed with 1X PBS. The pellet was resuspended in 100 μl Annexin-V-Fluos labeling solution (10 μl annexin reagent and 10 μl PI solution in 150 μl incubation buffer (according to manufacturer, Roche). The samples were incubated for 7 min in the dark, at room temperature then 100 μl incubation buffer was added. The cellular fluorescence was then measured using a Fluorescence Activated Cell Sorter (FACS) flow cytometer (BDFACS CantoTM II). Each sample was collected as 20,000 ungated events and the different cell populations were determined using FlowJo software (FlowJo7.6.5).

### Immunohistochemistry

Immunohistochemistry (IHC) was performed on available paraffin fixed tissue blocks from a xenograft experiment [10] to detect the expression of pERK1/2. Rehydration of tissue sections was performed in descending concentrations solutions of ethanol (96% to 70%). Antigen was retrieved by heating in a pressure cooker (1 mmol/L Tris-EDTA buffer, 120°C, 5 min). The slices were incubated in blocking solution (Dako, Glostrup, Denmark) to prevent nonspecific binding sites. Next pERK1/2 (1:2000) primary antibody was added to the slices and incubated for 30 min at room temperature. The sections were then washed with washing buffer (Dako) and incubated with EnVision + System horseradish peroxidase-linked secondary antibody (goat anti-rabbit, Dako) at room temperature for 30 min. Positive immunoreactivity was detected using diaminobenzidine + (Dako). Positive and negative IHC controls were included in this study. Percentage and intensity of positively stained epithelial cells (cytoplasmic versus nuclei) was quantified and scored by an expert pathologist (T.T.R.). Statistical analysis was performed using SPSS. Two tailed student t-test was performed by comparing 2 samples assuming that they have equal variances.

## Electronic supplementary material

Additional file 1: Table S1: Known TQ targets identified by the peptide array. (DOCX 105 KB)

Additional file 2: Figure S1: TQ did not induce changes in the main kinases affected by IPA-3. Cells treated with 40 μM TQ were collected after 1, 3, 6 and 24 hours. Untreated cells were used as a control. 30 μg of proteins per well were used to assess the activity of AKT2, GSKα, GSKβ and p38. Data is presented as fold activity normalized to control activity. Each value is the mean ± SD of two independent experiments each done in duplicates. (TIFF 99 KB)

Additional file 3: Figure S2: TQ show low toxicity on normal intestinal cells. HCT116 **(A)** and HCEC **(B)** cells were treated with different TQ concentrations (0-100 μM) for 24 hours to define the IC_50_ value. Cell viability was assessed by crystal violet staining. Data are presented as percentage of control. Each value is the mean ± SD of three independent experiments done in quadruplicates. (TIFF 118 KB)

Additional file 4: Figure S3: IPA-3 and TQ bind to the autoinhibited dimer conformation of PAK1. **A.** Model of IPA-3 bound autoinhibited dimer conformation of PAK1 (PDB id:1kx5). Autoregulatory region (blue and green) and kinase domain (pink and blue) of PAK1 dimer are shown. IPA-3 is shown in red using a stick representation and the residues interacting with IPA-3 labeled in black. **B.** Model of IPA-3 and TQ bound autoinhibited dimer conformation of PAK1. TQ is shown in purple as a stick representation. The residues interacting with TQ are labeled in black. The rendering follows color legend used in **A.** (TIFF 5 MB)

Additional file 5: Table S2: PAK1 residues interference with TQ and/or IPA-3. (DOCX 15 KB)

Additional file 6: Figure S4: TQ induces down regulation of prosurvival pERK1/2 *in vivo*. Mouse xenograft experiment showing **A.** Immunohistochemical detection of pERK1/2 on tumors of control (left) and TQ treated (right) animals. **B.** Box plot analysis of the percentage of tumor cells expressing cytoplasmic pERK1/2 after TQ treatment. n represents the number of investigated mouse tissues in each group. *16 corresponds to sample 16 being an outlayer. (TIFF 1 MB)

Additional file 7: Figure S5: TQ induces the phosphorylation of pPAK1^Thr212^ before the phosphorylation of pERK1/2. Cells treated with 60 μM TQ were collected after 10, 15, 30, 45, 60 and 90 min. untreated cells were used as control. 30 μg of proteins were immunoblotted against pPAKThr212 and pERK1/2. GAPDH was used as loading control. Data shown are representative of two independent experiments. (TIFF 154 KB)

Additional file 8: Table S3: Hydrogen bonds formed at the PAK1 catalytic site in the absence and presence of TQ. (DOCX 18 KB)

## Abbreviations

TQ: Thymoquinone
AKT/PKB: Protein kinase B
ERK: Extracellular-signal-regulated kinase
Plk1 PBD: Serine/threonine kinase Polo-like kinase 1
PAK1: p21 protein (Cdc42/Rac)-activated kinase 1
IPA-3: 1,1′-disulfanediyldinaphthalen-2-ol.
