# Supplementary material for: Thymoquinone-induced conformational changes of PAK1 interrupt prosurvival MEK-ERK signaling in colorectal cancer
Source: Mol Cancer. 2014 Aug 29;13:201. doi: 10.1186/1476-4598-13-201 (PMC4158125; doi:10.1186/1476-4598-13-201)
Supplement: Supplementary file 1 — Additional file 1: Table S1: Known TQ targets identified by the peptide array. (DOCX 105 KB) [file 12943_2014_1399_MOESM1_ESM.docx]

**Table S1 Known TQ targets identified by the peptide array**

| PROTEIN | PROT_ID | FOLDS (24h) | FOLDS (48h) | DISEASE and TRAITS | LITERATURE |
| --- | --- | --- | --- | --- | --- |
| AKT1 | NP_005154 | 2,22 |  | human umbilical vein endothelial cell HUVEC | [[1](#_ENREF_1)] |
|  |  |  |  | maturation of dendritic cells | [[2](#_ENREF_2)] |
|  |  |  |  | breast cancer | [[3](#_ENREF_3), [4](#_ENREF_4)] |
|  |  |  |  | primary effusion lymphoma (PEL) cell lines | [[5](#_ENREF_5)] |
|  |  |  |  | multiple myeloma | [[6](#_ENREF_6)] |
|  |  |  |  | lung (LNM35), liver (HepG2), colon (HT29), melanoma (MDA-MB-435), and breast (MDA-MB-231 and MCF-7) tumors | [[4](#_ENREF_4)] |
|  |  |  |  | skin cancer (squamous cell carcinoma in vitro and sarcoma 180-induced tumors in vivo) | [[7](#_ENREF_7)] |
|  |  |  |  | hepatic fibrosis | [[8](#_ENREF_8)] |
| Glucocorticoid receptor | NP_000167 | 2,15 |  | hepatic drug metabolizing | [[9](#_ENREF_9)] |
| Nitric oxide synthase 1 | NP_000611 | 2,09 |  | Endothelial dysfunction | [[10](#_ENREF_10)] |
|  |  |  |  | lung injury after chronic toluene exposure | [[11](#_ENREF_11)] |
|  |  |  |  | spermatogenesis after testicular injury | [[12](#_ENREF_12)] |
|  |  |  |  | hypertension and renal damage | [[13](#_ENREF_13)] |
|  |  |  |  | morphine tolerance and dependence | [[14](#_ENREF_14)] |
|  |  |  |  | pyrogallol-induced endothelial dysfunction | [[15](#_ENREF_15)] |
|  |  |  |  | inflammatory changes associating asthma | [[16](#_ENREF_16)] |
|  |  |  |  | renal hypertrophy | [[17](#_ENREF_17)] |
|  |  |  |  | diabetes mellitus | [[18](#_ENREF_18)] |
|  |  |  |  | immunomodulatory role of thymoquinone | [[19](#_ENREF_19)] |
| JAK2 | NP_004963 | 2,07 | 1,45 | multiple myeloma | [[20](#_ENREF_20)] |
| p21Cip1 | NP_510867 | 1,96 |  | breast cancer | Formularbeginn  [[3](#_ENREF_3)] Formularende |
|  |  |  |  | colorectal cancer | [[21](#_ENREF_21)] |
|  |  |  |  | prostate cancer | [[22](#_ENREF_22)] |
| NFkB-p65 | NP_1138610 | 1,91 |  | osteoarthritis | [[23](#_ENREF_23)] |
|  |  |  |  | rheumatoid arthritis | [[24](#_ENREF_24)] |
|  |  |  |  | myeloid leukemia | [[25](#_ENREF_25)] |
|  |  |  |  | proinflammatory responses in lipopolysaccharide-activated mast cells | [[26](#_ENREF_26)] |
| JNK | NP_620637 | 1,90 |  | Colon cancer | [[27](#_ENREF_27)] |
| CD45 | NP_002829 | 1,80 |  | multiple myeloma | [[6](#_ENREF_6)] |
| PI3K, regulatory subunit, alpha | P27986 | 1,80 | 1,43 | liver fibrosis | [[8](#_ENREF_8)] |
|  |  |  |  | blood platelets | [[28](#_ENREF_28)] |
| p27Kip1 | NP_004055 | 1,69 | 1,60 | prostate cancer | [[22](#_ENREF_22)] |
| c-Myc | NP_002458 | 1,69 |  | myeloid leukemia | [[25](#_ENREF_25)] |
| p53 | NP_000537 | 1,58 | 1,78 | breast cancer | [[3](#_ENREF_3), [29](#_ENREF_29)] |
|  |  |  |  | cervical squamous carcinoma | [[30](#_ENREF_30)] |
|  |  |  |  | glioblastoma cells | [[31](#_ENREF_31)] |
|  |  |  |  | colorectal cancer | [[21](#_ENREF_21), [32](#_ENREF_32), [33](#_ENREF_33)] |
|  |  |  |  | osteosarcoma | [[33](#_ENREF_33)] |
|  |  |  |  | skin tumorigenesis | [[34](#_ENREF_34)] |
| HMG CoA reductase | NP_000850 | 1,48 |  | cardiovascular disease | [[35](#_ENREF_35)] |
|  |  |  |  | cholesterol | [[36](#_ENREF_36)] |
| IKK beta | AAD08997 |  | 2,44 | Formularbeginn  myeloid leukemiaFormularende | [[25](#_ENREF_25)] |
| ERK2 | NP_620407 |  | 1,43 | osteogenesis | [[37](#_ENREF_37)] |
|  |  |  |  | glioblastoma | [[38](#_ENREF_38)] |
|  |  |  |  | colon cancer | [[27](#_ENREF_27)] |

1. Yi T, Cho SG, Yi Z, Pang X, Rodriguez M, Wang Y, Sethi G, Aggarwal BB, Liu M: **Thymoquinone inhibits tumor angiogenesis and tumor growth through suppressing AKT and extracellular signal-regulated kinase signaling pathways**. *Molecular cancer therapeutics* 2008, **7**(7):1789-1796.

2. Xuan NT, Shumilina E, Qadri SM, Gotz F, Lang F: **Effect of thymoquinone on mouse dendritic cells**. *Cellular physiology and biochemistry : international journal of experimental cellular physiology, biochemistry, and pharmacology* 2010, **25**(2-3):307-314.

3. Arafa el SA, Zhu Q, Shah ZI, Wani G, Barakat BM, Racoma I, El-Mahdy MA, Wani AA: **Thymoquinone up-regulates PTEN expression and induces apoptosis in doxorubicin-resistant human breast cancer cells**. *Mutation research* 2011, **706**(1-2):28-35.

4. Attoub S, Sperandio O, Raza H, Arafat K, Al-Salam S, Al Sultan MA, Al Safi M, Takahashi T, Adem A: **Thymoquinone as an anticancer agent: evidence from inhibition of cancer cells viability and invasion in vitro and tumor growth in vivo**. *Fundamental & clinical pharmacology* 2012.

5. Hussain AR, Ahmed M, Ahmed S, Manogaran P, Platanias LC, Alvi SN, Al-Kuraya KS, Uddin S: **Thymoquinone suppresses growth and induces apoptosis via generation of reactive oxygen species in primary effusion lymphoma**. *Free radical biology & medicine* 2011, **50**(8):978-987.

6. Badr G, Lefevre EA, Mohany M: **Thymoquinone inhibits the CXCL12-induced chemotaxis of multiple myeloma cells and increases their susceptibility to Fas-mediated apoptosis**. *PloS one* 2011, **6**(9):e23741.

7. Das S, Dey KK, Dey G, Pal I, Majumder A, MaitiChoudhury S, kundu SC, Mandal M: **Antineoplastic and apoptotic potential of traditional medicines thymoquinone and diosgenin in squamous cell carcinoma**. *PloS one* 2012, **7**(10):e46641.

8. Bai T, Lian LH, Wu YL, Wan Y, Nan JX: **Thymoquinone attenuates liver fibrosis via PI3K and TLR4 signaling pathways in activated hepatic stellate cells**. *International immunopharmacology* 2013, **15**(2):275-281.

9. Elbarbry F, Ragheb A, Marfleet T, Shoker A: **Modulation of hepatic drug metabolizing enzymes by dietary doses of thymoquinone in female New Zealand White rabbits**. *Phytotherapy research : PTR* 2012, **26**(11):1726-1730.

10. Idris-Khodja N, Schini-Kerth V: **Thymoquinone improves aging-related endothelial dysfunction in the rat mesenteric artery**. *Naunyn-Schmiedeberg's archives of pharmacology* 2012, **385**(7):749-758.

11. Kanter M: **Thymoquinone attenuates lung injury induced by chronic toluene exposure in rats**. *Toxicology and industrial health* 2011, **27**(5):387-395.

12. Kanter M: **Thymoquinone reestablishes spermatogenesis after testicular injury caused by chronic toluene exposure in rats**. *Toxicology and industrial health* 2011, **27**(2):155-166.

13. Khattab MM, Nagi MN: **Thymoquinone supplementation attenuates hypertension and renal damage in nitric oxide deficient hypertensive rats**. *Phytotherapy research : PTR* 2007, **21**(5):410-414.

14. Abdel-Zaher AO, Mostafa MG, Farghly HM, Hamdy MM, Omran GA, Al-Shaibani NK: **Inhibition of brain oxidative stress and inducible nitric oxide synthase expression by thymoquinone attenuates the development of morphine tolerance and dependence in mice**. *European journal of pharmacology* 2013, **702**(1-3):62-70.

15. El-Agamy DS, Nader MA: **Attenuation of oxidative stress-induced vascular endothelial dysfunction by thymoquinone**. *Experimental biology and medicine (Maywood, NJ)* 2012, **237**(9):1032-1038.

16. Ammar el SM, Gameil NM, Shawky NM, Nader MA: **Comparative evaluation of anti-inflammatory properties of thymoquinone and curcumin using an asthmatic murine model**. *International immunopharmacology* 2011, **11**(12):2232-2236.

17. Kanter M: **Protective effects of thymoquinone on streptozotocin-induced diabetic nephropathy**. *Journal of molecular histology* 2009, **40**(2):107-115.

18. El-Mahmoudy A, Shimizu Y, Shiina T, Matsuyama H, El-Sayed M, Takewaki T: **Successful abrogation by thymoquinone against induction of diabetes mellitus with streptozotocin via nitric oxide inhibitory mechanism**. *International immunopharmacology* 2005, **5**(1):195-207.

19. El-Mahmoudy A, Matsuyama H, Borgan MA, Shimizu Y, El-Sayed MG, Minamoto N, Takewaki T: **Thymoquinone suppresses expression of inducible nitric oxide synthase in rat macrophages**. *International immunopharmacology* 2002, **2**(11):1603-1611.

20. Li F, Rajendran P, Sethi G: **Thymoquinone inhibits proliferation, induces apoptosis and chemosensitizes human multiple myeloma cells through suppression of signal transducer and activator of transcription 3 activation pathway**. *British journal of pharmacology* 2010, **161**(3):541-554.

21. Wirries A, Breyer S, Quint K, Schobert R, Ocker M: **Thymoquinone hydrazone derivatives cause cell cycle arrest in p53-competent colorectal cancer cells**. *Experimental and therapeutic medicine* 2010, **1**(2):369-375.

22. Kaseb AO, Chinnakannu K, Chen D, Sivanandam A, Tejwani S, Menon M, Dou QP, Reddy GP: **Androgen receptor and E2F-1 targeted thymoquinone therapy for hormone-refractory prostate cancer**. *Cancer research* 2007, **67**(16):7782-7788.

23. Chen WP, Tang JL, Bao JP, Wu LD: **Thymoquinone inhibits matrix metalloproteinase expression in rabbit chondrocytes and cartilage in experimental osteoarthritis**. *Experimental biology and medicine (Maywood, NJ)* 2010, **235**(12):1425-1431.

24. Vaillancourt F, Silva P, Shi Q, Fahmi H, Fernandes JC, Benderdour M: **Elucidation of molecular mechanisms underlying the protective effects of thymoquinone against rheumatoid arthritis**. *Journal of cellular biochemistry* 2011, **112**(1):107-117.

25. Sethi G, Ahn KS, Aggarwal BB: **Targeting nuclear factor-kappa B activation pathway by thymoquinone: role in suppression of antiapoptotic gene products and enhancement of apoptosis**. *Molecular cancer research : MCR* 2008, **6**(6):1059-1070.

26. El Gazzar MA, El Mezayen R, Nicolls MR, Dreskin SC: **Thymoquinone attenuates proinflammatory responses in lipopolysaccharide-activated mast cells by modulating NF-kappaB nuclear transactivation**. *Biochimica et biophysica acta* 2007, **1770**(4):556-564.

27. El-Najjar N, Chatila M, Moukadem H, Vuorela H, Ocker M, Gandesiri M, Schneider-Stock R, Gali-Muhtasib H: **Reactive oxygen species mediate thymoquinone-induced apoptosis and activate ERK and JNK signaling**. *Apoptosis : an international journal on programmed cell death* 2010, **15**(2):183-195.

28. Towhid ST, Schmidt EM, Schmid E, Munzer P, Qadri SM, Borst O, Lang F: **Thymoquinone-induced platelet apoptosis**. *Journal of cellular biochemistry* 2011, **112**(11):3112-3121.

29. Sutton KM, Doucette CD, Hoskin DW: **NADPH quinone oxidoreductase 1 mediates breast cancer cell resistance to thymoquinone-induced apoptosis**. *Biochemical and biophysical research communications* 2012, **426**(3):421-426.

30. Ng WK, Yazan LS, Ismail M: **Thymoquinone from Nigella sativa was more potent than cisplatin in eliminating of SiHa cells via apoptosis with down-regulation of Bcl-2 protein**. *Toxicology in vitro : an international journal published in association with BIBRA* 2011, **25**(7):1392-1398.

31. Cecarini V, Quassinti L, Di Blasio A, Bonfili L, Bramucci M, Lupidi G, Cuccioloni M, Mozzicafreddo M, Angeletti M, Eleuteri AM: **Effects of thymoquinone on isolated and cellular proteasomes**. *The FEBS journal* 2010, **277**(9):2128-2141.

32. Gali-Muhtasib H, Kuester D, Mawrin C, Bajbouj K, Diestel A, Ocker M, Habold C, Foltzer-Jourdainne C, Schoenfeld P, Peters B *et al*: **Thymoquinone triggers inactivation of the stress response pathway sensor CHEK1 and contributes to apoptosis in colorectal cancer cells**. *Cancer research* 2008, **68**(14):5609-5618.

33. Roepke M, Diestel A, Bajbouj K, Walluscheck D, Schonfeld P, Roessner A, Schneider-Stock R, Gali-Muhtasib H: **Lack of p53 augments thymoquinone-induced apoptosis and caspase activation in human osteosarcoma cells**. *Cancer biology & therapy* 2007, **6**(2):160-169.

34. Gali-Muhtasib HU, Abou Kheir WG, Kheir LA, Darwiche N, Crooks PA: **Molecular pathway for thymoquinone-induced cell-cycle arrest and apoptosis in neoplastic keratinocytes**. *Anti-cancer drugs* 2004, **15**(4):389-399.

35. Ahmad S, Beg ZH: **Hypolipidemic and antioxidant activities of thymoquinone and limonene in atherogenic suspension fed rats**. *Food chemistry* 2013, **138**(2-3):1116-1124.

36. Al-Naqeep G, Ismail M, Allaudin Z: **Regulation of low-density lipoprotein receptor and 3-hydroxy-3-methylglutaryl coenzyme A reductase gene expression by thymoquinone-rich fraction and thymoquinone in HepG2 cells**. *Journal of nutrigenetics and nutrigenomics* 2009, **2**(4-5):163-172.

37. Wirries A, Schubert AK, Zimmermann R, Jabari S, Ruchholtz S, El-Najjar N: **Thymoquinone accelerates osteoblast differentiation and activates bone morphogenetic protein-2 and ERK pathway**. *International immunopharmacology* 2013, **15**(2):381-386.

38. Kolli-Bouhafs K, Boukhari A, Abusnina A, Velot E, Gies JP, Lugnier C, Ronde P: **Thymoquinone reduces migration and invasion of human glioblastoma cells associated with FAK, MMP-2 and MMP-9 down-regulation**. *Investigational new drugs* 2012, **30**(6):2121-2131.
