# Supplementary figures and images for: Thymoquinone-induced conformational changes of PAK1 interrupt prosurvival MEK-ERK signaling in colorectal cancer
Source: Mol Cancer. 2014 Aug 29;13:201. doi: 10.1186/1476-4598-13-201 (PMC4158125; doi:10.1186/1476-4598-13-201)

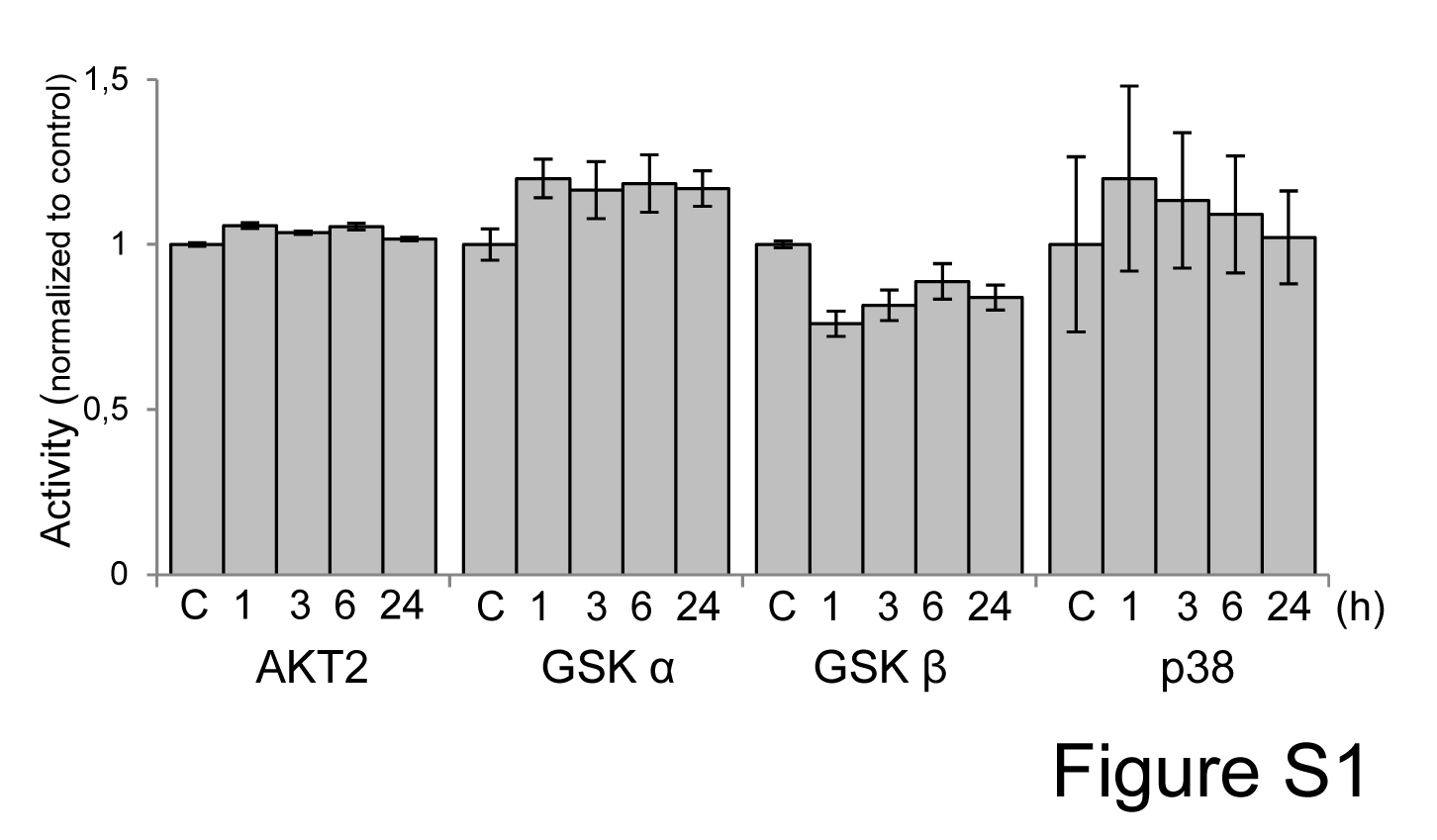

Supplement: Supplementary file 2 — Additional file 2: Figure S1: TQ did not induce changes in the main kinases affected by IPA-3. Cells treated with 40 μM TQ were collected after 1, 3, 6 and 24 hours. Untreated cells were used as a control. 30 μg of proteins per well were used to assess the activity of AKT2, GSKα, GSKβ and p38. Data is presented as fold activity normalized to control activity. Each value is the mean ± SD of two independent experiments each done in duplicates. (TIFF 99 KB) [file 12943_2014_1399_MOESM2_ESM.tiff]

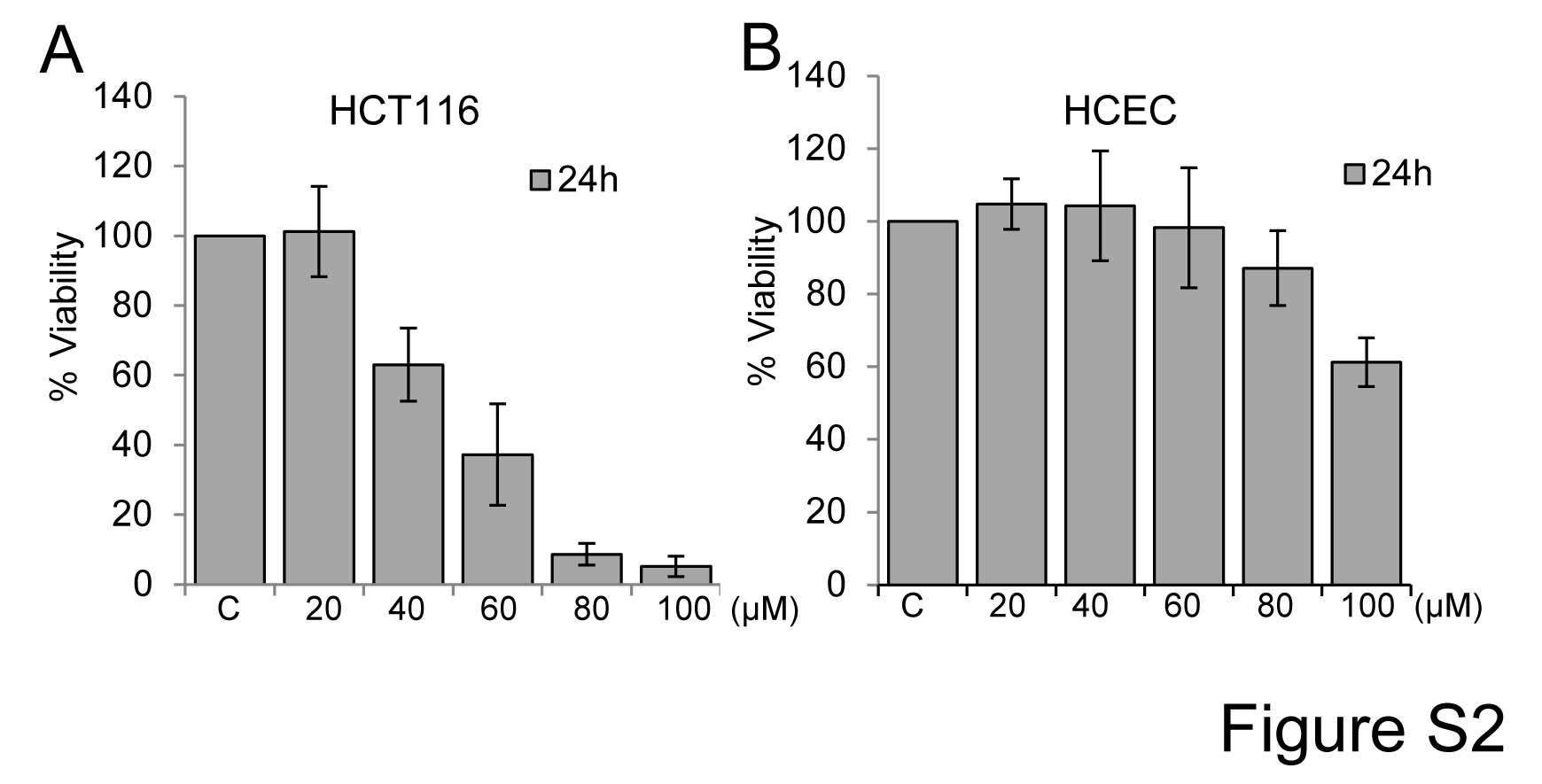

Supplement: Supplementary file 3 — Additional file 3: Figure S2: TQ show low toxicity on normal intestinal cells. HCT116 (A) and HCEC (B) cells were treated with different TQ concentrations (0-100 μM) for 24 hours to define the IC50 value. Cell viability was assessed by crystal violet staining. Data are presented as percentage of control. Each value is the mean ± SD of three independent experiments done in quadruplicates. (TIFF 118 KB) [file 12943_2014_1399_MOESM3_ESM.tiff]

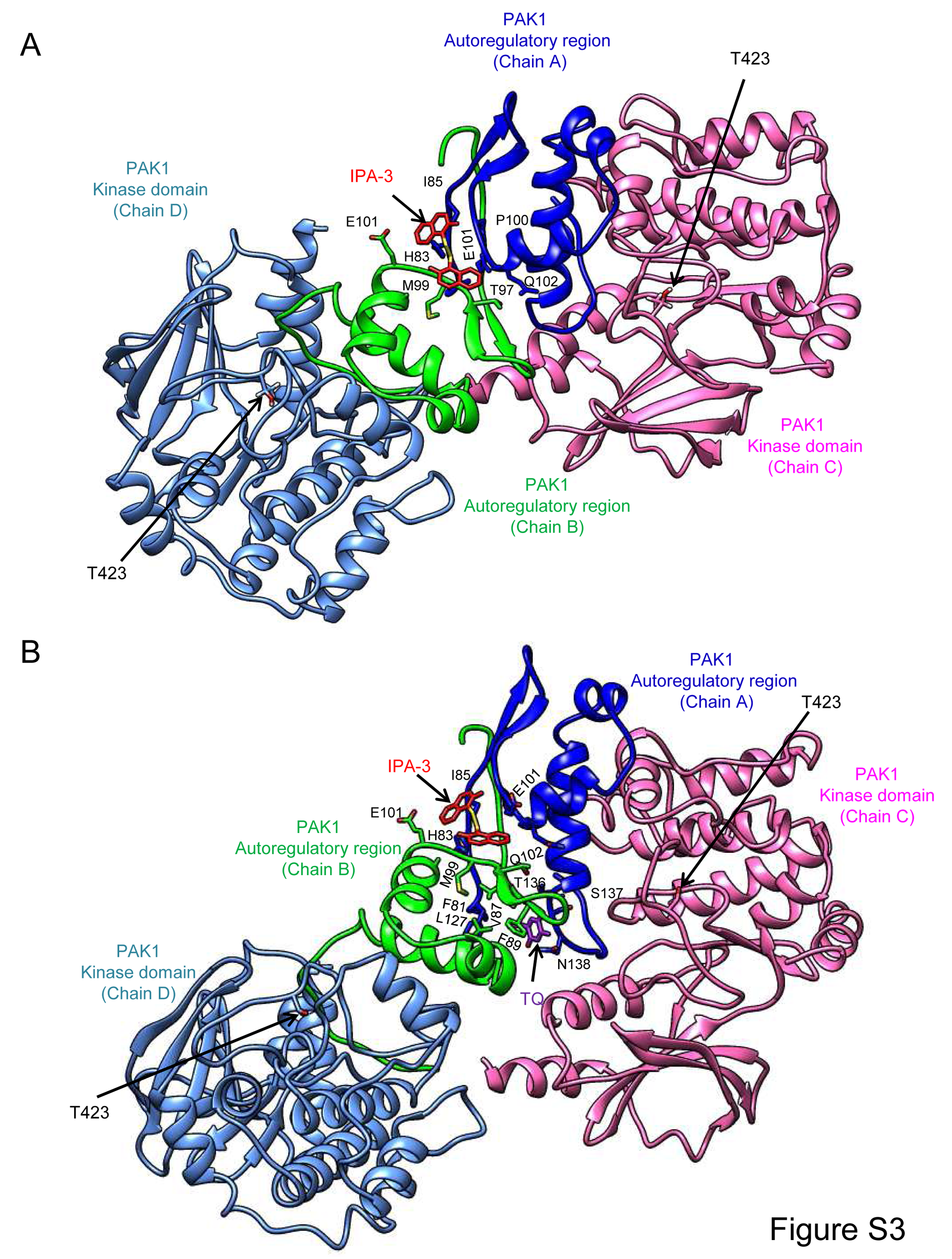

Supplement: Supplementary file 4 — Additional file 4: Figure S3: IPA-3 and TQ bind to the autoinhibited dimer conformation of PAK1. A. Model of IPA-3 bound autoinhibited dimer conformation of PAK1 (PDB id:1kx5). Autoregulatory region (blue and green) and kinase domain (pink and blue) of PAK1 dimer are shown. IPA-3 is shown in red using a stick representation and the residues interacting with IPA-3 labeled in black. B. Model of IPA-3 and TQ bound autoinhibited dimer conformation of PAK1. TQ is shown in purple as a stick representation. The residues interacting with TQ are labeled in black. The rendering follows color legend used in A. (TIFF 5 MB) [file 12943_2014_1399_MOESM4_ESM.tiff]

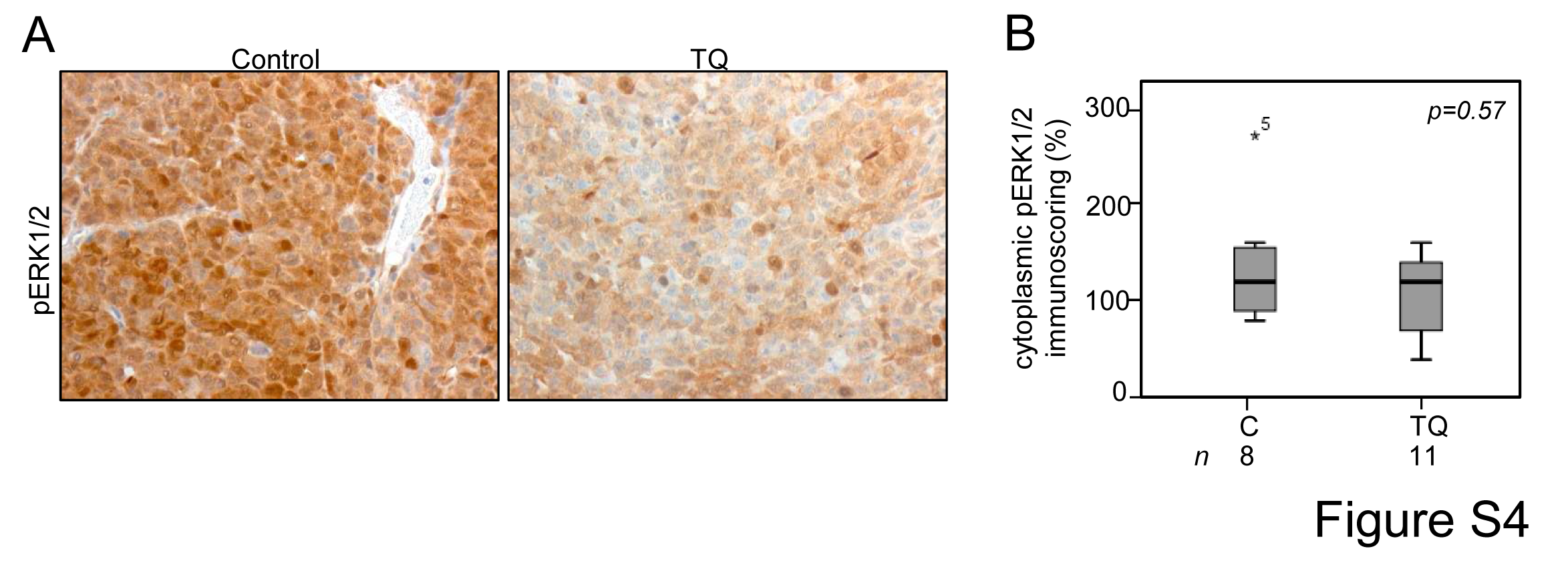

Supplement: Supplementary file 6 — Additional file 6: Figure S4: TQ induces down regulation of prosurvival pERK1/2 in vivo. Mouse xenograft experiment showing A. Immunohistochemical detection of pERK1/2 on tumors of control (left) and TQ treated (right) animals. B. Box plot analysis of the percentage of tumor cells expressing cytoplasmic pERK1/2 after TQ treatment. n represents the number of investigated mouse tissues in each group. *16 corresponds to sample 16 being an outlayer. (TIFF 1 MB) [file 12943_2014_1399_MOESM6_ESM.tiff]

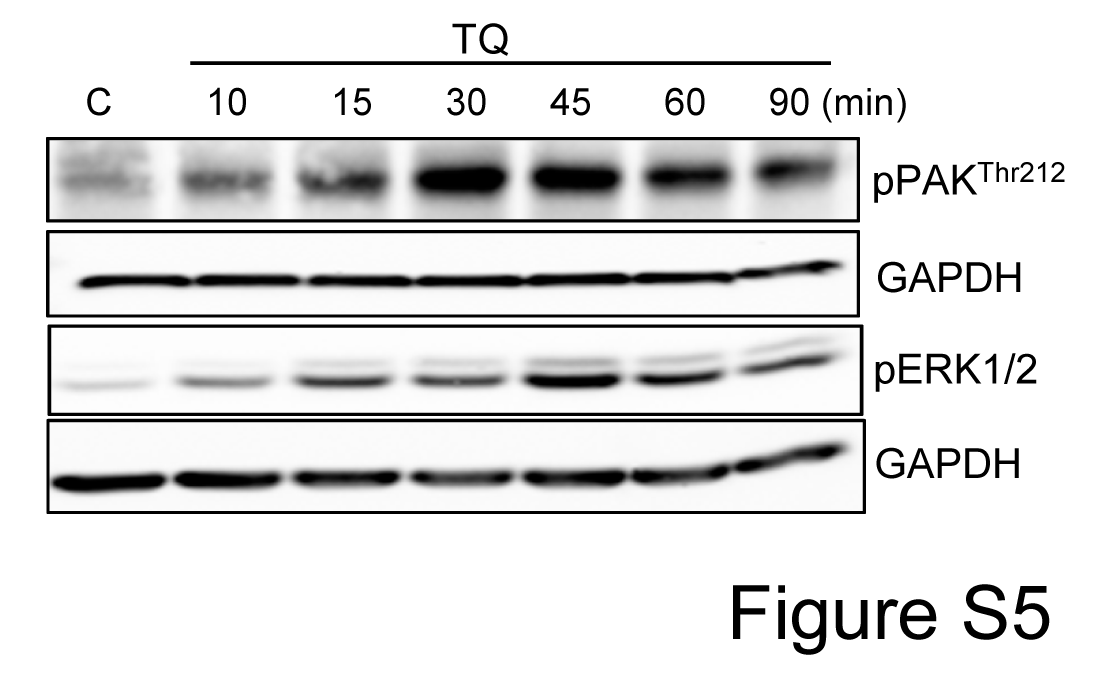

Supplement: Supplementary file 7 — Additional file 7: Figure S5: TQ induces the phosphorylation of pPAK1Thr212 before the phosphorylation of pERK1/2. Cells treated with 60 μM TQ were collected after 10, 15, 30, 45, 60 and 90 min. untreated cells were used as control. 30 μg of proteins were immunoblotted against pPAKThr212 and pERK1/2. GAPDH was used as loading control. Data shown are representative of two independent experiments. (TIFF 154 KB) [file 12943_2014_1399_MOESM7_ESM.tiff]
