# Supplementary material for: Thymoquinone-induced conformational changes of PAK1 interrupt prosurvival MEK-ERK signaling in colorectal cancer
Source: Mol Cancer. 2014 Aug 29;13:201. doi: 10.1186/1476-4598-13-201 (PMC4158125; doi:10.1186/1476-4598-13-201)
Supplement: Supplementary file 5 — Additional file 5: Table S2: PAK1 residues interference with TQ and/or IPA-3. (DOCX 15 KB) [file 12943_2014_1399_MOESM5_ESM.docx]

**Table S2 PAK1 residues interference with TQ and/ or IPA-3**

| **PAK1 conformation** | **Ligand** | **PAK1 residues at the 4Å interface of ligand binding** | **Glide Docking Score** |
| --- | --- | --- | --- |
| PAK1 active state conformation | TQ | Thr 214, Ala 218, Pro 221, Val 217, Thr 212, Val 211, Pro 210, Leu 209 | -2.316 |
| Autoinhibited conformation of PAK1(PDB id: 1F3M) | IPA-3 | A: His 83, Gly 98, Met 99, Val 87, Pro 100, Ile 85, His 86, Gln 102, Trp 103, Glu 101  B:Met 99, Gly 98, Thr 97, Phe 96, Ala 104, Pro100, Glu 101 | -4.407 |
| Inhibited conformation of PAK1 by IPA-3 | TQ | A: Asn 138, Phe 81, Ser 137, Thr136,  B: Phe 89 , Gly 88, His86, Gln 122, Val 87, Leu 125 | -3.574 |
