# Supplementary material for: Thymoquinone-induced conformational changes of PAK1 interrupt prosurvival MEK-ERK signaling in colorectal cancer
Source: Mol Cancer. 2014 Aug 29;13:201. doi: 10.1186/1476-4598-13-201 (PMC4158125; doi:10.1186/1476-4598-13-201)
Supplement: Supplementary file 8 — Additional file 8: Table S3: Hydrogen bonds formed at the PAK1 catalytic site in the absence and presence of TQ. (DOCX 18 KB) [file 12943_2014_1399_MOESM8_ESM.docx]

**Table S3** Hydrogen bonds formed at the PAK1 catalytic site in the absence and presence of TQ

| **PAK1** |  |  |  | **PAK1-TQ** |  |  |
| --- | --- | --- | --- | --- | --- | --- |
| **Residue 1** | **Residue 2** | **Hbond distance(Å)** |  | **Residue 1** | **Residue 2** | **Hbond distance(Å)** |
| R388 NE | F410 O | 2.88 |  | R388 NE | F410 O | 2.96 |
| R388 NH2 | F410 O | 3.29 |  |  |  |  |
| R388 NH1 | Y441 OH | 2.9 |  | R388 NH1 | Y441 OH | 2.7 |
| R388 N | D446 OD1 | 2.94 |  | R388 N | D446 OD1 | 2.84 |
|  |  |  |  | R388N | D446 OD2 | 3.29 |
| D389 N | H387 O | 3.27 |  | D389 N | H387 O | 3.12 |
| D389 N | H387 ND1 | 2.51 |  |  |  |  |
| D389 OD2 | N394 ND2 | 3.42 |  | D389 OD2 | N394 ND2 | 2.78 |
| K391 N | D389 O | 3.45 |  | K391 N | D389 O | 2.3 |
|  |  |  |  | K391 NZ | D389 OD2 | 2.53 |
| E417 N | T415 O | 3.38 |  | E417 N | T415 O | 3.3 |
| Q418 N | T415 O | 3.26 |  | Q418 N | T415 O | 3.5 |
| S419 N | T415 O | 3.01 |  | S419 N | T415 O | 3.02 |
|  |  |  |  | S419 N | E417 O | 2.78 |
| K420 N | Q418 O | 3.49 |  | K420 N | Q418 O | 3.15 |
| V425 N | T423 O | 3.36 |  | V425 N | T423 O | 3.1 |
| Q418 N | T415 OG1 | 3.25 |  |  |  |  |
| Q418 NE2 | K420 O | 2.88 |  | Q418 NE2 | K420 O | 2.88 |
|  |  |  |  | S419 OG | E417 O | 3.1 |
| R421 NH2 | Q413 O | 2.93 |  | R421 NH2 | Q413 O | 2.8 |
|  |  |  |  | R421 NE | Q413 O | 3 |
| T415 OG1 | E417 OE1 | 3.4 |  |  |  |  |
| T415 OG1 | E417 OE2 | 3.13 |  | T415 OG1 | E417 OE2 | 2.6 |
| V425 N | T423 O | 3.36 |  | V425 N | T423 O | 3.1 |
| T427 N | D389 OD1 | 3.03 |  | T427 N | D389 OD1 | 2.73 |
| T427 OG1 | D389 OD1 | 2.77 |  | T427 OG1 | D389 OD1 | 2.58 |
|  |  |  |  | T427 OG1 | D389 OD2 | 3.46 |
| Y441 N | R421 O | 3.01 |  | Y441 N | R421 O | 2.97 |
| Y441 OH | T423 O | 2.69 |  | Y441 OH | T423 O | 2.63 |
